# Telecommuting and employees’ mental health

**DOI:** 10.1192/j.eurpsy.2021.924

**Published:** 2021-08-13

**Authors:** M.D.C. Molina Lietor, I. Cuevas, M. Blanco Prieto

**Affiliations:** Psiquiatría, Hospital Universitario Príncipe de Asturias, Alcalá de Henares, Spain

**Keywords:** TELECOMMUTING, mental health, Covid, pandemic

## Abstract

**Introduction:**

Telecommuting is defined as “a work practice that involves members of an organization substituting a portion of their typical work hours to work away from a central workplace, using technology to interact with others as needed to conduct work tasks”. The prevalence rate of telecommuting in Sapin in 2019 was 5%, while this rate grew up to 34% during de COVID-19 pandemic.

**Objectives:**

The purpose of this poster is to make a review about how telecommuting affects the employees’ mental health.

**Methods:**

A review os the available literature on employees’ menthal health.

**Results:**

Most employers who offer telecommuting consider it a strategic decision for their businesses: it could reduce the expenses of physically accommodating the employees, and it might help employers to contact their subordinates anytime, if needed. Many articles in the popular press about telecommuting extol the benefits of this practice on employees’ health (work-life balance, reduction of travel expenses). However, only a handful of empirical studies substantiate these claims (job satisfaction, quality of life, and role-related stress). Less discussed is the potential of telecommuting to have a negative impact on employees’ health. It may increase both social and professional isolation, which in turn is associated with higher levels of emotional exhaustion, cynicism, cognitive stress complaints and lower levels of work engagement.

**Conclusions:**

Finally, although it is not as effective as personal contact, organizations may stimulate qualitative virtual interaction with coworkers by providing robust online meeting tools and infrastructure, so users can seamlessly collaborate regardless of their physical location.

